# Creation of 3D Model of Stainless-Steel Billet’s Grain after Three-High Screw Rolling

**DOI:** 10.3390/ma15030995

**Published:** 2022-01-27

**Authors:** Mikhail Mikhailovich Skripalenko, Stanislav Olegovich Rogachev, Boris Alekseevich Romantsev, Sergei Pavlovich Galkin, Liudmila Mikhailovna Kaputkina, Mikhail Nikolaevich Skripalenko, Andrei Vladimirovich Danilin, Viktor Aleksandrovich Fadeev

**Affiliations:** 1 Department of Metal Forming, National University of Science and Technology “MISiS”, Leninski Prospect, 4, 119049 Moscow, Russia; boralr@yandex.ru (B.A.R.); glk-omd@yandex.ru (S.P.G.); kaputkina@mail.ru (L.M.K.); tfsmn@yandex.ru (M.N.S.); danilinav@yandex.ru (A.V.D.); fdv_viktor@mail.ru (V.A.F.); 2 Department of Physical Metallurgy and Physics of Strength, National University of Science and Technology “MISiS”, Leninski Prospect, 4, 119049 Moscow, Russia; csaap@mail.ru

**Keywords:** screw rolling, three-high rolling mill, billet, grain, 3D model, stainless steel

## Abstract

The three-high screw rolling of AISI 321 billet from 60 mm to 52 mm diameter was performed using an MISIS-100T mill. When screw rolling was carried out, a set of sections were made in the billet’s cross-section at the stationary stage of screw rolling. SolidWorks was applied to make the 3D model of the rolled billet’s grain using microstructure images. The same technique was applied for the creation of the 3D model of a nondeformed billet’s grain. A comparison of the 3D models’ shape and dimensions before and after screw rolling was made. It was established that, compared to the nondeformed grain model, the screw rolled billet’s grain model was twisted and elongated along some angle in the rolling direction. This angle’s value is commensurable to the roll’s feed angle during the experimental rolling. Anisotropy indexes of before and after rolling grain models were estimated and compared to the anisotropy indexes obtained via the sections’ analysis in earlier research. Difference did not exceed 5%.

## 1. Introduction

Screw rolling is applied for manufacturing round bars, hollow tube shells and seamless tubes. Two-high rolling mills are mainly used for producing hollow tube shells by screw piercing [1,2,3,4,5,6,7,8]. In some cases, three-high screw piercing is used for the same purpose [8,9,10]. Three-high screw rolling is applied for manufacturing round bars [11,12,13,14]. It can be noticed that, during screw rolling, the material forming process is complex in character [15]. The microstructure is often investigated and estimated while screw rolling. This estimation and investigation are mainly performed for the cross-section at the stationary stage of rolling [10,16,17,18,19,20]. The forming character during screw rolling certainly influences microstructure formation. For instance, the variation of the anisotropy index at the stationary stage of three-high screw rolling was shown in [21]. It is shown in [21] that the stitched structure of the rolled billet’s longitudinal section is 15–17 degrees oriented in the rolling direction. This means that the feed angle of rolls was 18 degrees. Similar results were obtained for the microstructure sections in the billet’s longitudinal section close to the billet’s surface [22]. It was observed in [19,23] that grains are oriented with some angle in the areas near the billet’s surface in the cross-section. The orientation of the grains at some angle was observed in the center of the A 516-55 steel billet after three-high radial-shear rolling in [24]. This appears to actually provide the three-dimensional visualization of rolled metal grain, i.e., the creation of a grain 3D model. Due to the complexity of metal flow during screw rolling [15,21], using 2D (flat) microstructure images cannot fully reflect the way in which the whole grain shape is changed. It can only tell what happens with a cross or longitudinal section of the grain, without giving the exact value of grain dimensions. 3D visualization will provide a more detailed understanding of how screw rolling influences formed metal microstructure formation. The identification of grain formation at screw rolling using the 3D approach will provide wider opportunities (compared to the microstructure data obtained using flat microstructure sections) for the clarification of existing notions concerning the billet’s stress–strain state while screw rolling, including the confirmation of the helical flow character of the billet’s material [25]. According to our review, there are no published techniques for making 3D grain models during screw rolling.

The objective of this research was the development of a technique for the creation of a 3D model of AISI 321 steel billet’s grain before and after three-high screw rolling. It was also the objective to compare the created 3D models to estimate how three-high screw rolling influences the grain’s shape and dimensions.

## 2. Materials and Methods

### 2.1. Experimental Three-High Screw Rolling

The screw rolling of AISI 321 steel (NUST “MISiS”, Moscow, Russian) billets was performed using a MISIS-100T three-high rolling mill (The Electrostal Heavy EngineeringWorks JSC (“EZTM” JSC), Electrostal city, Moscow Region, Russian Federation). The initial billet was manufactured by longitudinal rolling and had a 60 mm diameter and 200 mm length. The billet was heated until 1150 °C and kept in the furnace at this temperature for 2 h before screw rolling. After that billet was immediately placed in the mill for rolling, hence, there was no intermediate cooling (the billet was rolled in homogenized condition). The grain size of the initial billet ranged from 35 μm in the center of the billet to 125 μm on the surface of the billet [21]. The hardness of the initial billet ranged from 116 HV in the center of the billet to 122 HV on the surface of the billet [21]. The chemical composition of the steel of the rolled billet is presented in Table 1. Diameter after screw rolling was 52 mm. Billet was cooled in water after screw rolling. Feed angle of rolls was 18 degrees, inclination angle of rolls was 10 degrees and angular velocity of rolls was 5.76 rad/s.

### 2.2. Creation of 3D Model of Grain after Three-High Screw Rolling

**Figure 1:**
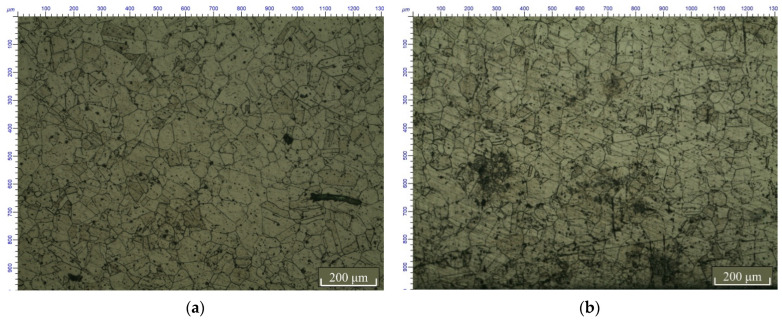
Billet’s microstructure 5 mm from the surface in the plane perpendicular to the rolling axis (**a**) and in the plane parallel to the rolling axis (**b**).

**Figure 2:**
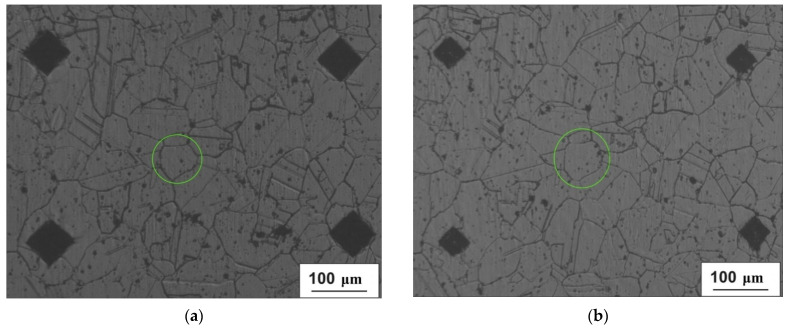

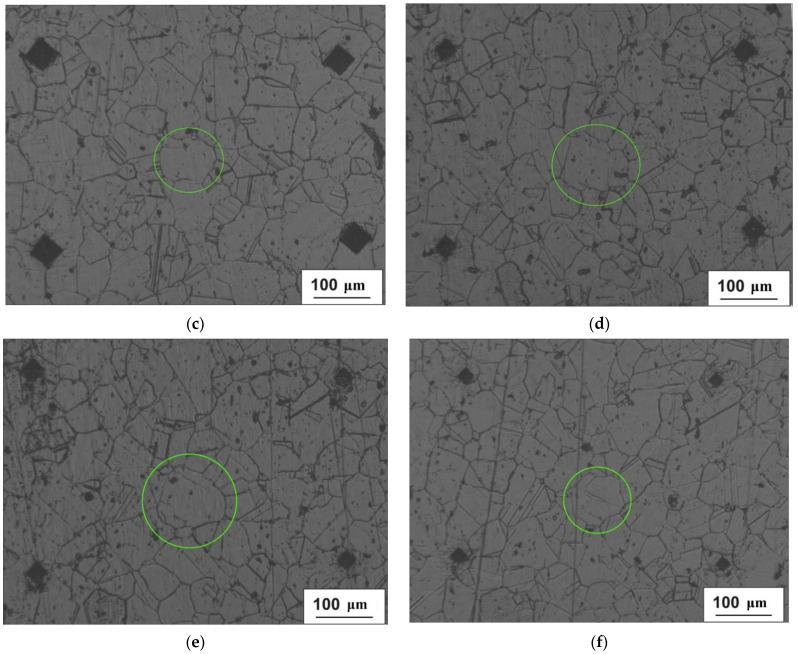
Sections with the same grain contours used for making the grain 3D model: (**a**)—section #1; (**b**)—section #2; (**c**)—section #3; (**d**)—section #4; €—section #5; and (**f**)—section #6.

**Figure 3:**
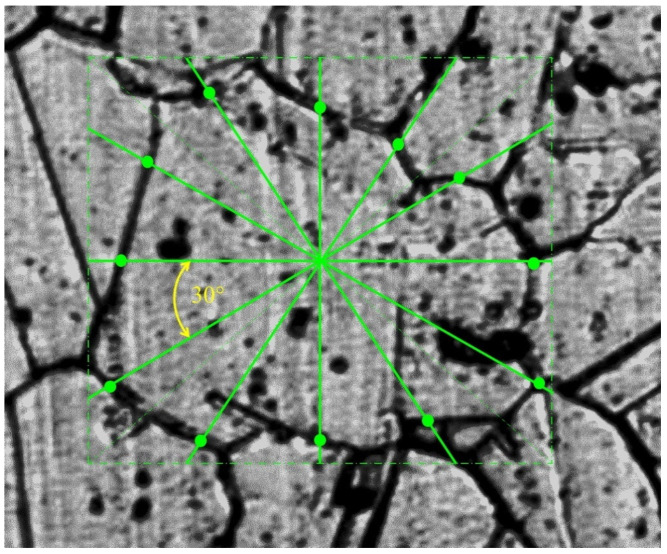
Twelve points on the grain contour.

**Figure 4:**
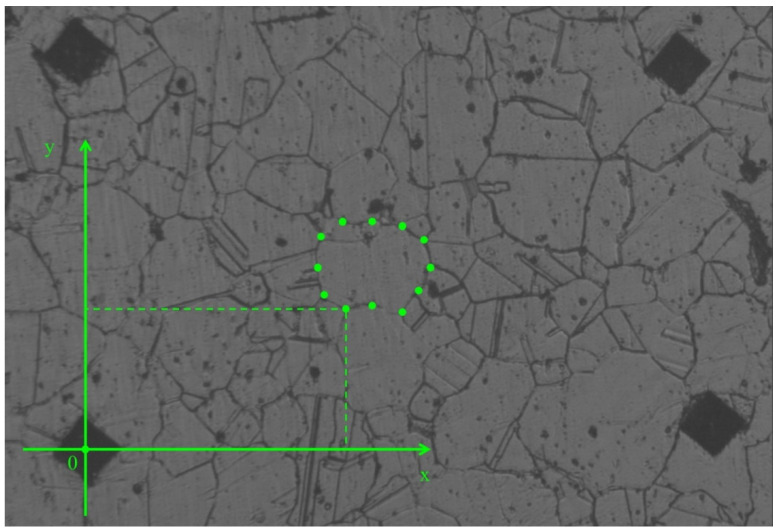
Origin and location of the points of the section with respect to the origin.

**Figure 5:**
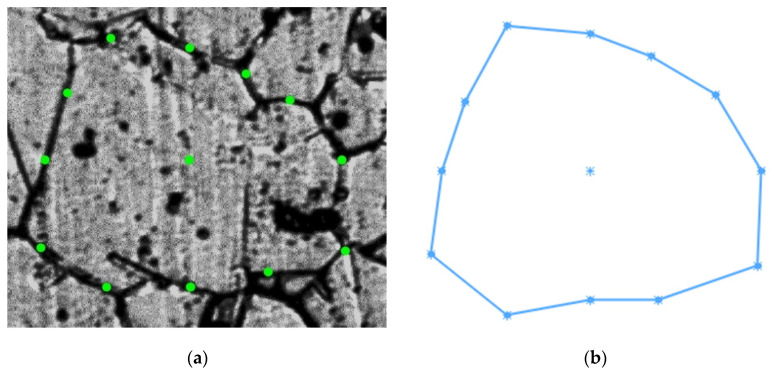
Cross-section of the grain (**a**) and its contour as a polygon sketch in SolidWorks (**b**).

**Figure 6:**
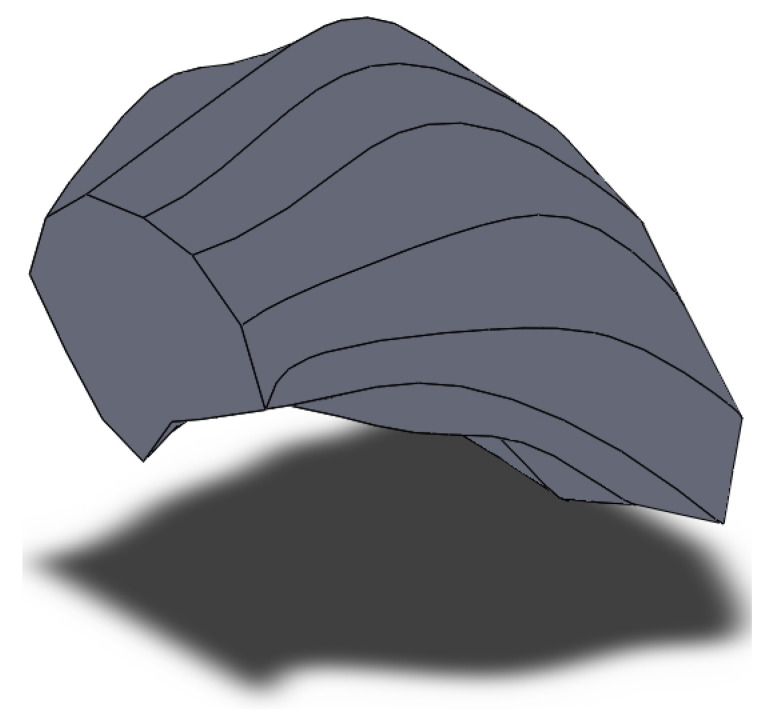
3D model of grain 5 mm from the billet’s surface area after three-high screw rolling.

Grain 3D model was designed for the area located 5 mm from the billet’s surface and at the stationary stage of rolling. Two primary sections were made in interperpendicular planes. One of the sections was made in the plane perpendicular to the rolling axis, and another one in the plane parallel to rolling axis. Maximum grain size was estimated in each of the sections. The estimation of the maximum grain size value for the section at Figure 1a (perpendicular to rolling axis) was 150 μm. The estimation of maximum grain size for the section parallel to the rolling axis (Figure 1b) was 130 μm. The largest of the two estimations was 150 μm. Eleven secondary sections were made in planes perpendicular to the rolling axis and hence, parallel to the plane of one of the primary sections. The distance between the first and eleventh section was 150 μm, i.e., corresponding to an estimation of the maximum grain size. The distance between the adjacent sections was from 5 to 25 μm. Contours (cross-sections) corresponding to the same grain were sought in each section. Grain whose contours are seen on 6 sections (Figure 2) was identified. The distance between the first and sixth section was 113 μm. Grains contours were reproduced in SolidWorks. Twelve points were fixed at each microstructure photograph. These points were on the straight lines and the angle between adjacent lines was 30 degrees (Figure 3). All straight lines passed through the point which was the center of the rectangle described around the grain contour (Figure 3). While preparing section #1 (Figure 2a), pyramid indenters were pressed in and the prints of indenters were that deep so their prints were seen on each of the 6 sections. Center of the left lower print (Figure 4) of each section was taken as origin. Taking that into account, each of the 12 points had their own coordinates. Grain contours at each of the 6 sections were created in SolidWorks by 12 points. Additionally, the center of each contour was added in SolidWorks sketch. Points were joined with lines and polygons were obtained (Figure 5). In total, 6 sketches with polygons were created in parallel planes. Distance between these planes equaled distance between corresponding microstructure sections’ planes. The 3D grain model was made by applying the “Lofted Boss/Base” command of the “Features” supplementary sheet in the SolidWorks menu using 6 sketches (Figure 6).

### 2.3. Creation of 3D Model of Grain before Three-High Screw Rolling

**Figure 7:**
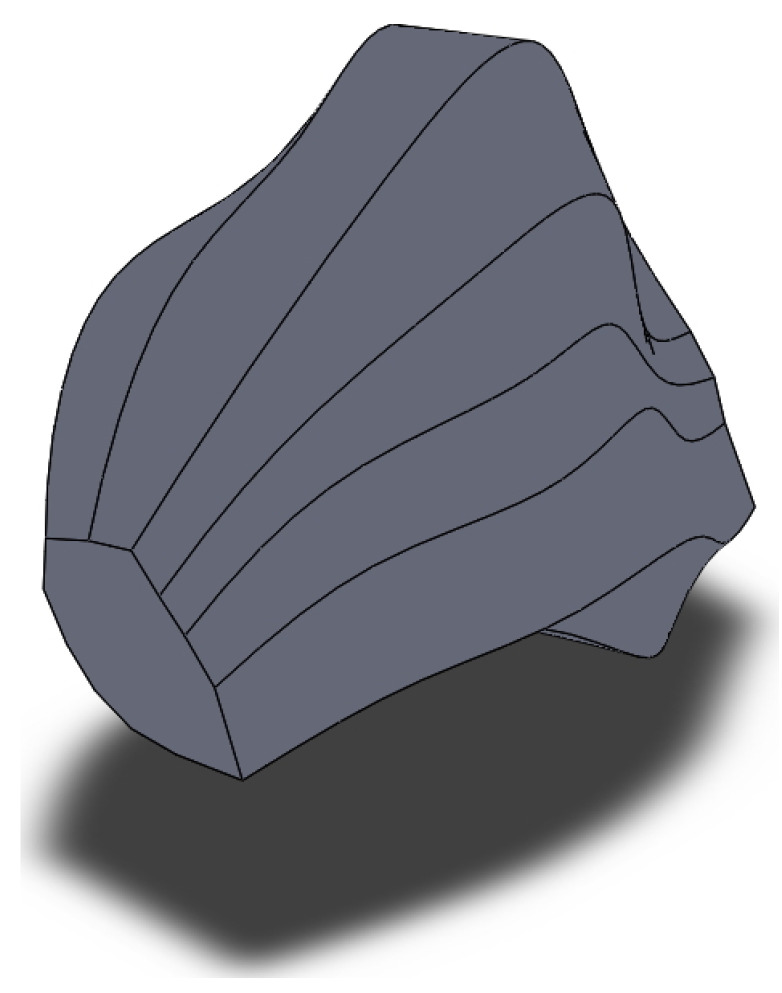
3D model of grain for 6 mm from the billet’s surface area before three-high screw rolling.

Using the technique described in the previous section, the 3D model of the grain of the initial billet (before screw rolling) was made. The grain 3D model was designed for the area, located 6 mm from the billet’s surface. Two primary microstructure sections in interperpendicular planes were made. The estimation of the maximum grain size in the section perpendicular to the rolling axis was 250 μm, and 270 μm for the section parallel to the rolling axis. Twenty-five microstructure sections in parallel planes were made on the 270 μm length. The planes of the microstructure sections were perpendicular to the rolling axis. One of the grains had contours at each of the sections. Six sections were chosen out of 25. These 6 sections were enough to make the 3D model of grain and to demonstrate tendencies of grain size changing (increasing grain size or decreasing grain size). Designed model is presented in Figure 7.

## 3. Discussion of Results

**Figure 8:**
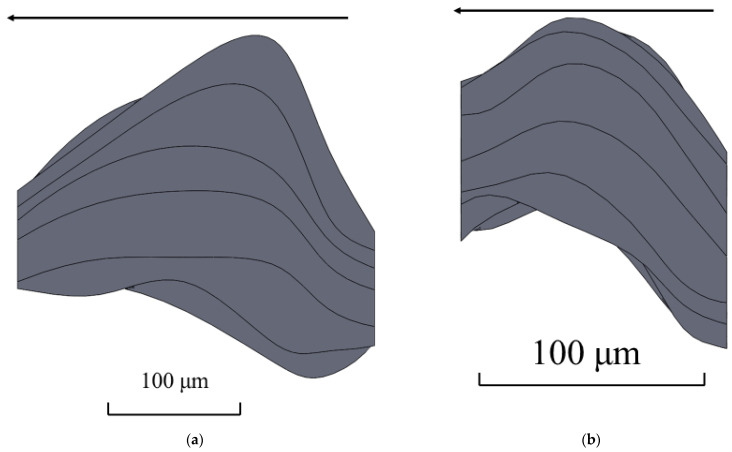

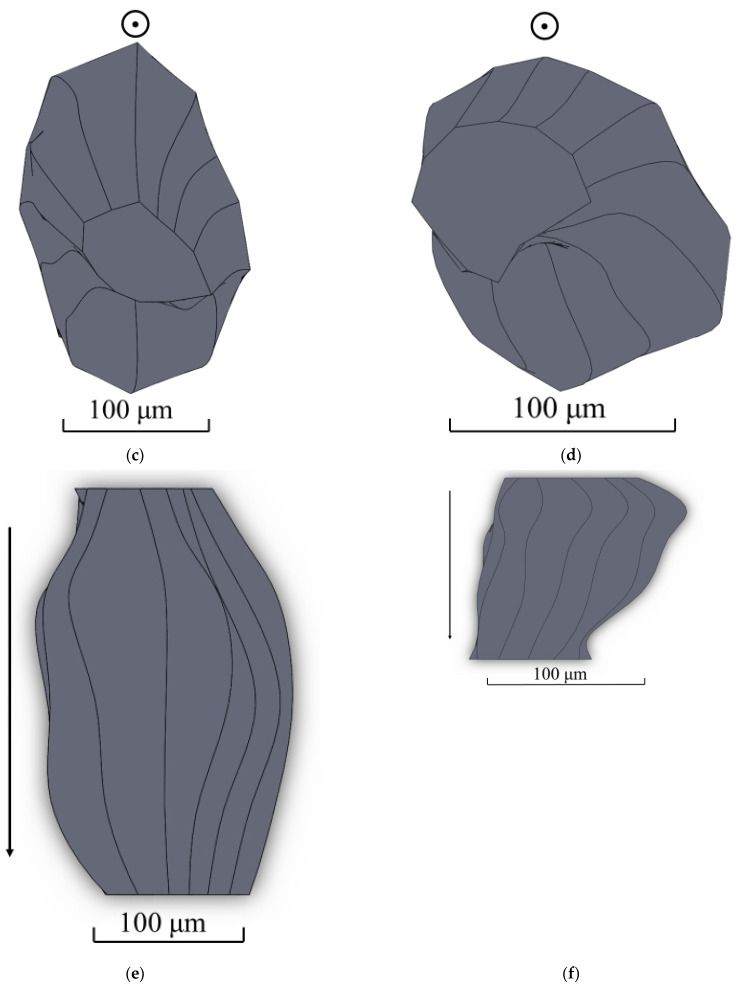
Side view (**a**), front view (**c**) and top view (**e**) of the grain 3D model before screw rolling and side view (**b**), front view (**d**) and top view (**f**) of grain 3D model after screw rolling (arrows identify the rolling direction).

Top, side and front views of each of the models were made using SolidWorks for the estimation of screw rolling influence on the shape and dimensions of the grain (Figure 8).

The 3D models presented in Figure 6, Figure 7 and Figure 8 allowed to conclude that there is twisting of the grain near the billet surface areas. This twisting means a change in the grain’s shape in the horizontal and vertical direction. The grain model before screw rolling is clearly elongated in one direction. This is due to initial billet which was manufactured by longitudinal rolling [20]. Designed 3D models also allow the estimation of grain size anisotropy. According to the measurement performed using SolidWorks tools (grain’s length was divided by grain’s thickness), the grain size anisotropy index is 1.19 for the initial billet and 0.95 for the screw rolled billet. In terms of the same screw rolling conditions [20], the estimation of the grain size anisotropy index for initial billet was 1.25 and 0.97 for the screw rolled billet. According to Figure 8, the grain size decreased up to 2.3 times which correlates with the decreasing grain size (2.2 times) estimated in earlier research [21] using linear intercept method. The hardness thus increased until 135–137 HV [21].

**Figure 9:**
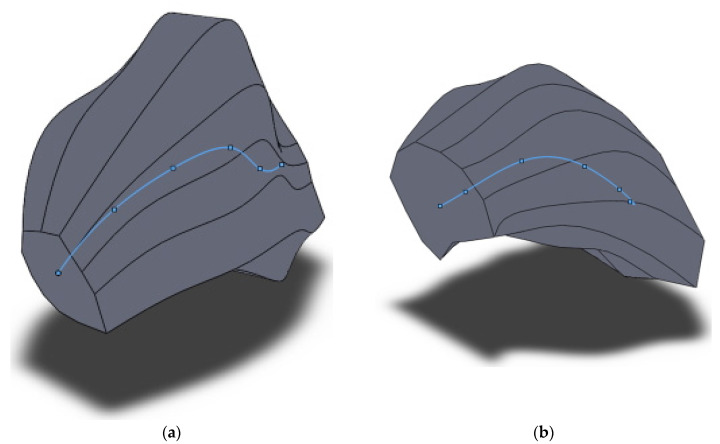
Splines passing through the centers of the models of grain before (**a**) and after (**b**) screw rolling.

**Figure 10:**
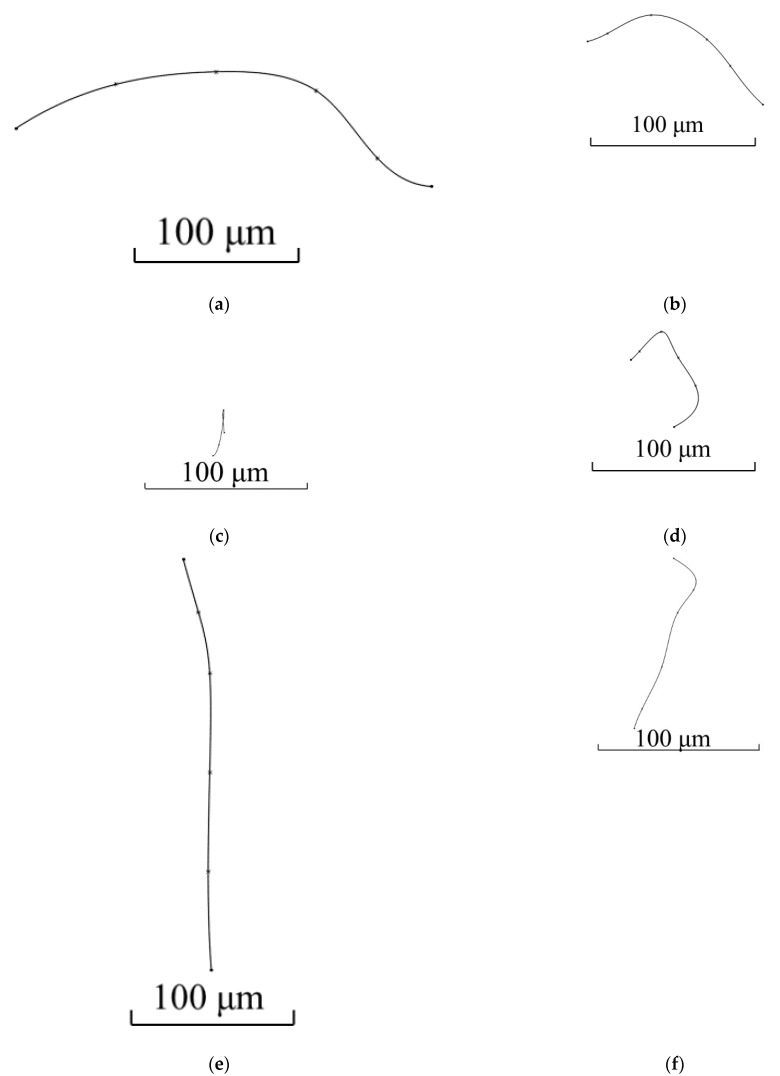
Spline passing through the cross-sections’ centers of the 3D model of grain before screw rolling (**a**,**c**,**e**) and spline passing through the cross-sections’ centers of the 3D model of grain after screw rolling (**b**,**d**,**f**): side views (**a**,**b**); front views (**c**,**d**); top views (**e**,**f**).

Lines joining the centers of each of the 6 cross-sections (polygons in sketches, used for obtaining 3D models) were created to enable the further qualitative and quantitative estimation of designed 3D models. The center of each cross-section was the point which was the center of the rectangle described around the grain contour (Figure 3). A three-dimensional sketch was created in SolidWorks, and then a spline was applied to join all the cross-section centers to obtain the line (curve) passing through the center of each cross-section of the model. The position of the spline inside each 3D model and three views of the spline for each of the models are shown in Figure 9 and Figure 10.

Figure 9 and Figure 10 allow the visual estimation of several changes: the maximum distance between cross-sections centers by height, width, and length of the 3D model of grain before screw rolling differ from the same parameters for the 3D model of grain after screw rolling. The maximum distance between the cross-section centers of the 3D grain models by height, width, and length before and after screw rolling were quantitatively compared (Table 2). The maximum distance by width increased 2.14 times after screw rolling. The maximum distance by height decreased 1.3 times after screw rolling. The maximum distance by length decreased 2.4 times after screw rolling. The spline’s shape in Figure 10d shows that the centers of the cross-sections form close to a half-circle contour. Analyzing Figure 10d,f, one can notice that half of the spline resembles helical line. Differences in the views of 3D models and splines clearly demonstrate this thesis concerning the microstructure twisting and helical type of material flow during three-high screw rolling.

**Figure 11:**
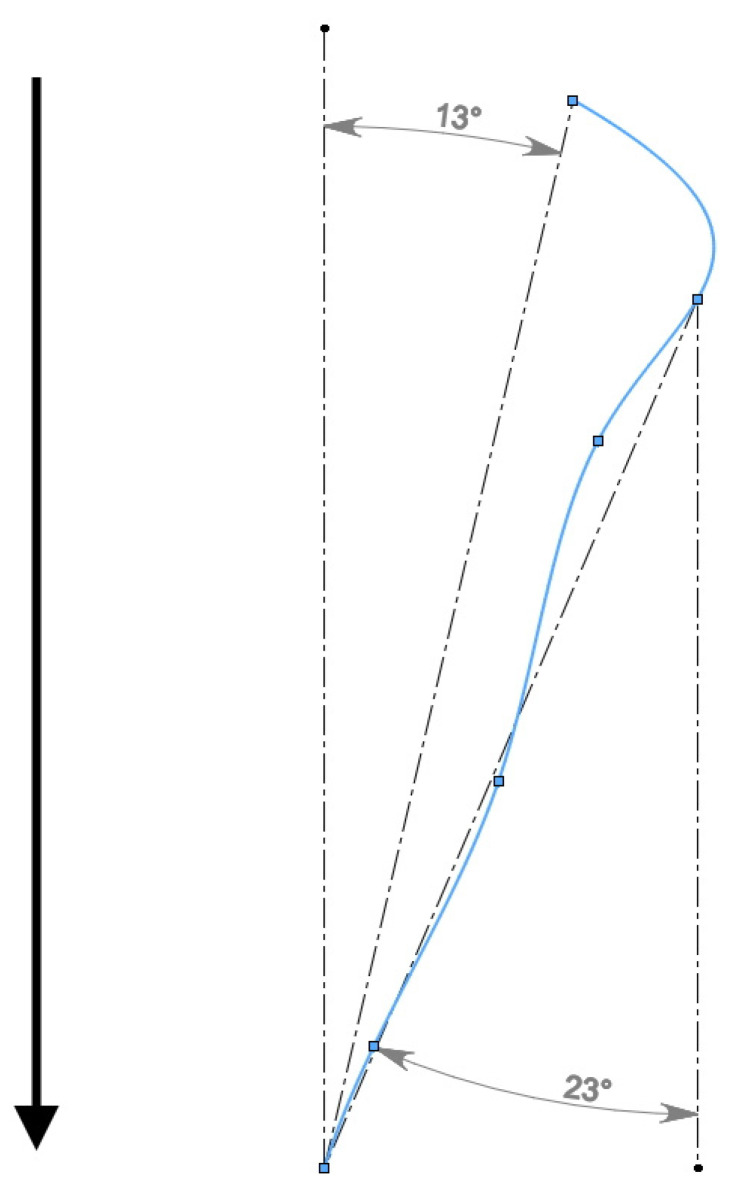
Estimation of the angle of grain orientation with respect to rolling axis (rolling direction is identified with arrow).

The top view of the grain 3D model after screw rolling (Figure 8e) shows that the grain is oriented at some angle towards the rolling axis. Using the spline for this model and this spline’s top view, the angle of grain orientation to the rolling axis was estimated (Figure 11).

According to Figure 11, the estimation of the angle between the grain long axis and screw rolling direction is within 13–23°. The feed angle of the rolls of the three-high screw rolling mill was 18° during experiments. It is worth noting that in terms of investigation [20], where rolling conditions were the same as for this research, stitched structure was 15–17° oriented with respect to the screw rolling axis. Hence, the designed 3D model of grain allows concluding that grains at screw rolling are oriented with angles close to the feed angles of rolls.

## 4. Conclusions

The three-high screw rolling of the AISI 321 steel billet was carried out. The estimation of the grain size was performed based on the microstructure investigation of the initial and rolled billet. The method for designing the 3D model of grain was developed. Using the developed method, 3D models of grains of initial and screw rolled billet were designed. At that, the grains for which models were made were located near to billet’s surface. According to the review, such 3D visualization was performed for the first time for screw rolling. Compared to existing techniques, which allow for the estimation of only the size of sections of the grain, the designed technique provides an estimation of all the grain three dimensions and visualizes all the changes in shape. Designed models allowed showing that the grain is initially elongated (oriented) in one direction, which is explained by the fact that the initial billet was manufactured by longitudinal rolling. The grain 3D model after rolling shows that the grain is twisted and elongated at some angle in the screw rolling direction. The value of this angle is commensurable with the value of the rolls’ feed angle. The anisotropy of the grains’ model shape is characterized by the anisotropy indexes’ value which differ by no more than 5% from the anisotropy indexes value before and after screw rolling, calculated based on flat microstructure sections’ investigation. The developed method can be used for designing a 3D model of grains for other metal forming processes and for an estimation of the influence of heat treatment on the materials’ microstructure, for instance, for studying recrystallization processes and grain growth. The interaction between different grains and the compatibility of forming a grain ensemble for keeping the material’s continuity should definitely be considered while making the model of transformation of deformable polycrystal object grain structure.

## Figures and Tables

**Table 1:** Chemical composition (in %) of the steel of the billet.

C	Si	Mn	Ni	S	P	Cr	Cu	Ti	Fe
≤0.12	≤0.8	≤2	9–11	≤0.02	≤0.035	17–19	≤0.3	0.4–1	~67

**Table 2:** Maximum distances between cross-sections of the grain models before and after three-high screw rolling.

	Maximum Distance between Cross-Section Centers, μm		
	By Width	By Height	By Length
Grain 3D model before screw rolling	18.4	74.8	270
Grain 3D model after screw rolling	39.5	57.9	113
